# The effect analysis of shape design of different charging piles based on Human physiological characteristics using the MF-DFA

**DOI:** 10.1038/s41598-024-59147-8

**Published:** 2024-04-09

**Authors:** Yusheng Zhang, Yaoyuan Kang, Xin Guo, Pan Li, Hanqing He

**Affiliations:** 1 Electric Power Research Institute of State Grid Shaanxi Electric Power Company, Xi’an, 710003 China; 2 State Grid Electric Auto Service Co., Ltd, Xi’an, 710003 China

**Keywords:** Charging pile, Shape design, MF-DFA, EEG, Psychology, Human behaviour, Neuroscience, Cognitive neuroscience

## Abstract

With the rapid development of new energy vehicles, the users have an increasing demand for charging piles. It is generally believed that the charging pile is a kind of practical product, and it only needs to realize the charging function. However, as a product, the shape design of the charging pile will directly affect the user experience, thus affecting product sales. Therefore, in the face of increasingly fierce market competition, when designing the shape of charging piles, it is necessary to adopt the traditional evaluation method and human physiological cognitive characteristics to evaluate the shape of charging piles more objectively. From the user’s point of view, using the electroencephalogram (EEG) of the user, with the help of the multifractal detrended fluctuation analysis (MF-DFA) method, this paper comprehensively analyzes the differences in emotional cognitive characteristics between two kinds of charging piles, namely, the charging pile with a curved appearance design and the charging pile with square appearance design. The results show that there are significant differences in human physiological cognitive characteristics between two kinds of charging piles with different shapes. And different shapes of charging piles have different physiological cognitive differences for users. When designing charging pile product shapes, human beings can objectively evaluate the product shape design according to the physiological cognition differences of users, so as to optimize the charging pile product shape design.

## Introduction

Product design represents a demand for aesthetic form, and human cognition of product appearance involves profound psychological processes^1–3^. Research has found that physiological signal features of the human body, such as electroencephalogram (EEG), electrooculogram (EOG), and electrocardiogram (ECG), can effectively reflect cognitive differences in product visual design^3–5^. Distinct EEG features are observed when individuals perceive different product appearances. Zhang Yanhe's research indicates significant cognitive differences among humans in perceiving products of different levels, such as high-end, mid-range, and low-end products, which can be effectively expressed through variations in human EEG voltage intensity^6^. Rus et al.’s study suggests that distinct cognitive differences arise from various object shapes, and these differences can be distinguished by analyzing EEG power spectral density^7^. Augustin et al. discovered that when people have different emotions to images, the corresponding EEG characteristics will show differences^3^. Furthermore, related experiments have found that human EOG features also vary according to different product appearances. Wang Zhenya’s findings demonstrate significant differences in eye movement trajectories and areas of visual fixation among subjects viewing cars with different designs^3^. Su Jianning's research results indicate that people exhibit distinct eye movement features, such as first gaze time, return time, and regression count, when observing products of different shapes^8^. Yan Longhua's study suggests that different clothing patterns lead to varying eye movement frequency characteristics in humans^9^. For diverse product appearances, human ECG features also exhibit notable differences. Zhang Shucheng et al.’s research suggests that when humans experience emotional changes due to product appearance, their heart rate characteristics undergo significant variations^10^.

In summary, given the significant cognitive differences exhibited by humans towards different product appearances, and considering the stability and ease of collection of electrooculogram (EOG)^11^ and electroencephalogram (EEG)^12^ signal features, this study employs human EOG and EEG features to analyze cognitive differences arising from two types of charging station designs. The study aims to determine whether there is a correlation between different charging station designs and human physiological signal features. Subsequently, the study aims to establish an evaluation method for charging station designs based on human EEG and EOG cognitive features. This method will then be applied to real-world design scenarios within the enterprise, facilitating better design and analysis of charging station shapes to align with people’s cognitive needs. Currently, charging station product design mainly involves two types: curved surface shape design (MDCSS) and square shape design (MDSSS). The former emphasizes curved surfaces, with characteristics such as smooth and gentle appearances. The latter highlights square shapes, characterized by simplicity and elegance. However, product design must meet the requirements of a wide range of consumers, which necessitates designers conducting extensive user research. Among various research methods, those based on human physiological cognitive features are relatively scarce, despite their strong objectivity. Thus, this experiment chooses human physiological features (EEG, EOG) to conduct comparative experiments on the external designs of MDCSS and MDSSS charging stations. Drawing on the experimental methods proposed by Sun Yuan et al., which combine eye-tracking data with the FAHP method for measuring product emotional cognition^5^; the EEG perception study of product appearance under the influence of brand identity by Zhang Yanhe et al.^6^; and the extraction of automotive design features and cognitive experiments based on eye-tracking technology conducted by Wang Zhenya et al.^3^, this study has been organized with the aim of achieving its objectives.

## Materials and methods

**Figure 1:**
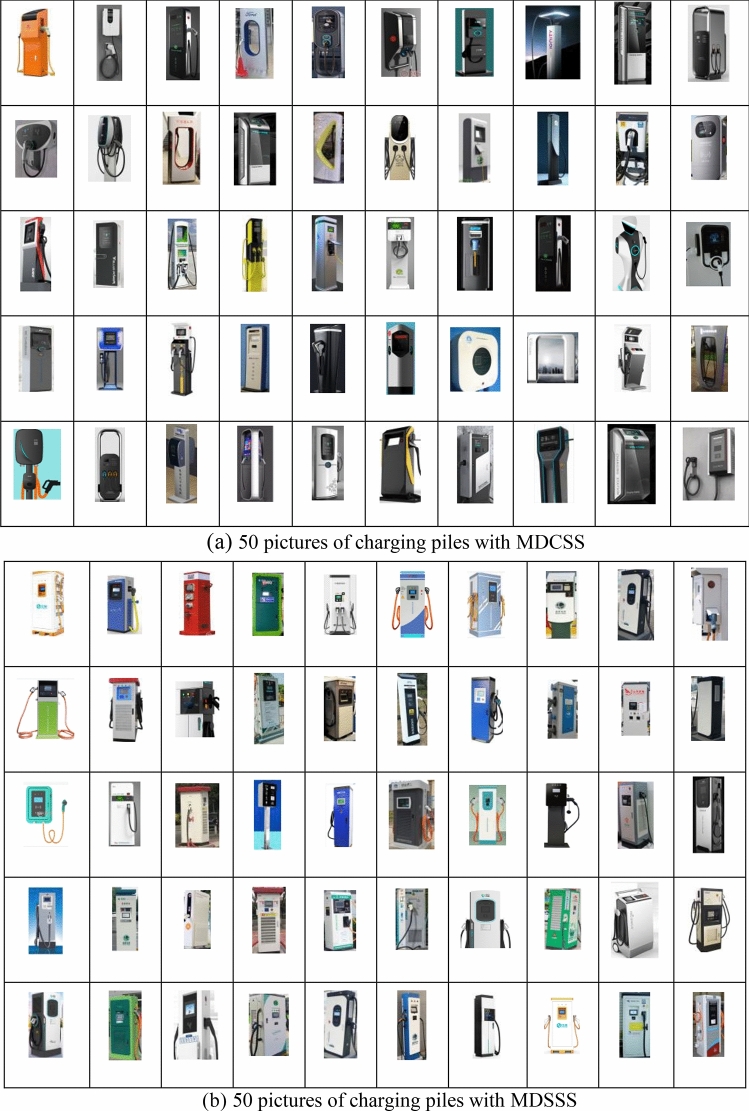
Pictures of two types of charging piles.

The physiological signal features of the human body can reflect cognitive differences in product exterior design^3–5^. To explore the differences in human cognition caused by different charging station designs, this study conducted experiments involving both the physiological information-based charging station exterior cognition and subjective survey questionnaires among users. In order to better investigate the cognitive differences caused by different charging station designs, volunteers with three years of experience driving electric vehicles were recruited. The research on human cognitive emotion classification has achieved fruitful results^13–16^. In these studies, 9^13^, 11^14^, 12^15^, and 20^16^ participants were selected, and these numbers of participants have been shown to effectively reflect the changes in different cognitive and emotional characteristics of humans. Therefore, this study selected 20 participants to participate in the experiment. The selected 20 participants were regular users of charging stations, with an average age of 33. The experiment was carried out in Xiangtan, China. The ratio of male drivers to female drivers in the region is about 4:1^17^. Therefore, in this study, we selected 16 male drivers and 4 female drivers to achieve a more objective evaluation of the shape of the charging pile. All participants had no history of mental illness or psychological disorders. Within 24 h before the experiment, participants refrained from consuming any stimulating beverages such as alcohol or coffee. For the experiment, a total of 50 images of MDSSS charging stations and 50 images of MDCSS charging stations were selected, as shown in Fig. 1. The names, models and manufacturers of the charging piles in Fig. 1 are detailed in Appendix I and Appendix II. The former (MDSSS) featured a traditional linear design with a square shell, while the latter (MDCSS) had a curved design with a multi-curved shell. During the experiment, portable electroencephalogram (EEG) recording equipment, the Emotiv, was used to collect EEG signals. The device had a sampling frequency of 128 Hz and was equipped with 14 EEG electrodes (Supplementary Information).

**Figure 2:**
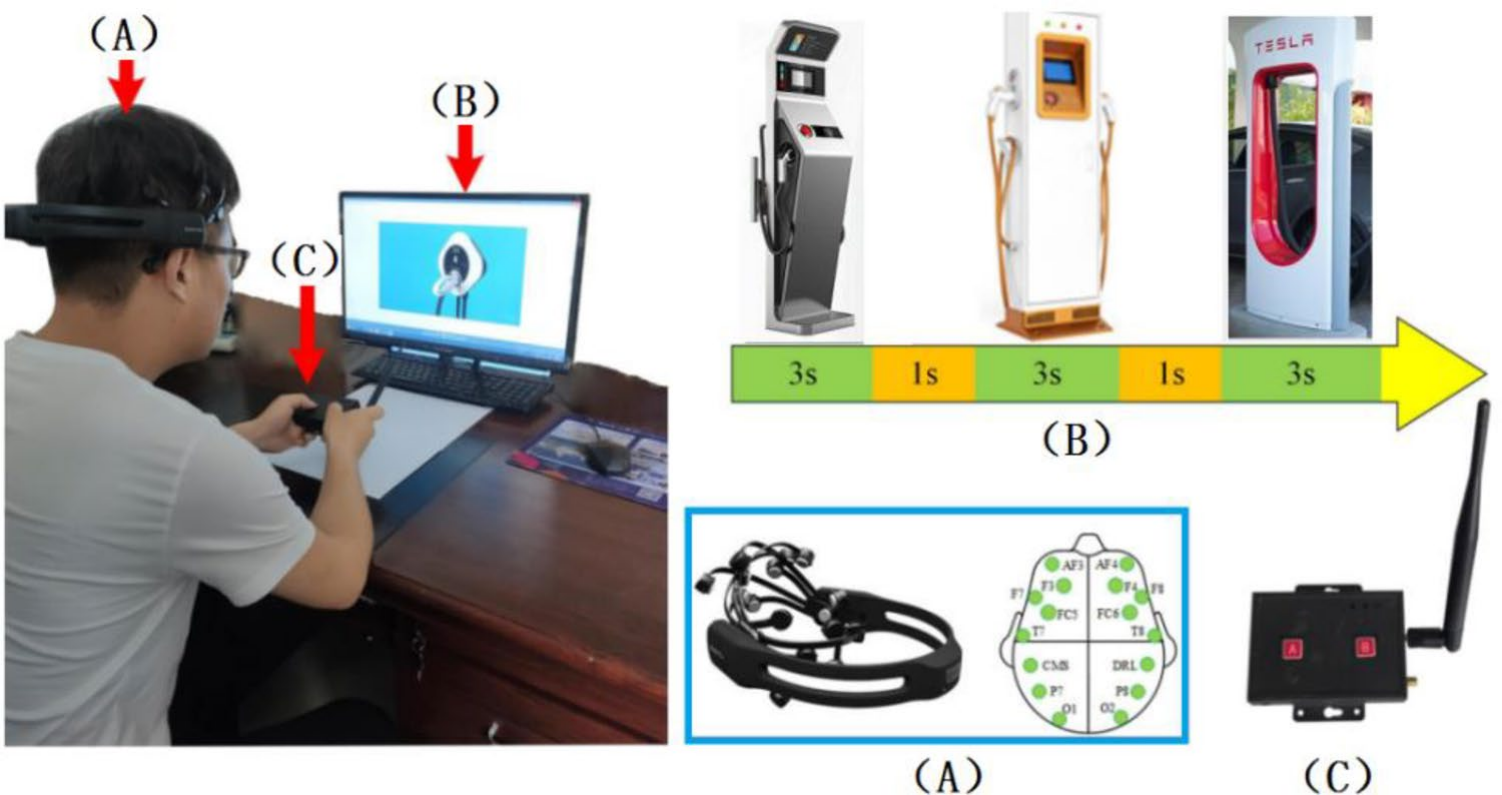
Experiment equipment.

All the charging station images had consistent heights, and the widths of the charging stations were matched to their respective heights. The charging station images were randomly arranged, with each image displayed on the screen for 3 s. During this time period, participants were required to provide subjective evaluations of the charging stations. If a participant liked the design, they pressed the button on the left side of the transceiver; otherwise, they pressed the button on the right side. The equipment is illustrated in Fig. 2.

According to the laboratory standards stipulated in GB/T4857.2-2005, the temperature of the laboratory was controlled in the range of 21–25℃, and the relative humidity was controlled in the range of 45–55%. In addition, participants should sit in a comfortable chair and keep their bodies relaxed, with their feet flat on the ground and their hands on a tabletop to ensure good posture and comfort. The computer monitor should be placed at eye level with a distance of 0.8–0.9 m from the participant's face. The lab uses soft light and tries to avoid harsh light sources to keep participants comfortable and focused. The noise was kept below 30 decibels to ensure it did not disturb the subjects.

### Data preprocessing

Due to the existence of noise signals such as electromagnetic signals in the experimental environment, it is necessary to preprocess the collected EEG signals for denoising before extracting the data features. In this study, wavelet packet decomposition was employed to denoise the EEG signals. The original EEG signals were decomposed into four levels, extracting the *θ* rhythm (4–8 Hz) and *β* rhythm (12–32 Hz) from the signals.

### Algorithm

Research has demonstrated that EEG signal features can reflect changes in human emotions^18–20^. When a person exhibits a liking emotion, the *α* waves in the occipital area EEG signals and the *β* waves in the central area EEG signals are activated, while the *θ* waves are suppressed^18^. In this study, we investigated the brain network features of EEG signals corresponding to leads in the occipital and central areas to analyze different emotional characteristics in humans. The main information features of brain signals are concentrated in the 0–32 Hz range, which can be divided into *δ*, *θ*, *α*, and *β* sub-bands based on frequency. The *β* sub-band typically appears when individuals are drowsy, lack focus, and other similar states, indicating an inhibitory state of the central nervous system. The *β* sub-band is associated with awake consciousness and reflects the activity of cortical cells in various intellectual activities related to logical thinking, reasoning, and problem-solving, which typically occurs during sensory input to the cortex and the operation of cortical neurons. Previous studies have shown that when humans are in a state of lack of focus, mental fatigue, or indifference to external behavior, the *θ* rhythm signals in EEG significantly increase, while the *β* rhythm signals decrease noticeably^21^. The MF-DFA method has strong anti-interference when dealing with nonlinear and non-stationary signals. This method can reveal the multifractal structure in the signal more comprehensively, which helps to capture complex features in EEG signals. Compared with traditional frequency domain and time domain analysis methods, MF-DFA is more suitable for processing nonlinear and non-stationary biological signals, and has better performance in extracting the emotional cognitive features of charging piles with different shapes^22^. Therefore, in the study, the MF-DFA algorithm was used to calculate the generalized Hurst exponent and multifractal spectrum corresponding to *θ* and *β* rhythm signals, and their features were analyzed. An introduction to the MF-DFA algorithm is provided below.

Assuming a non-stationary time series $\{ x_{k} \} \;(k = 1,\;2,\;3, \ldots ,\;n)$ of length *n*, the procedure to transform it into a random walk time series and calculate the fractal exponent is as follows:

(1) Calculate the mean of the time series using the formula (1):

1 $$\overline{x} = \frac{1}{n}\sum\limits_{k}^{n} {x_{k} }$$

(2) Calculate the cumulative deviation (i.e., the time series minus the mean) using the formula (2):

2 $$Y(i) = \sum\limits_{k = 1}^{i} {[x_{k} - \overline{x}]}$$

Based on the above processing, the noisy time series is transformed into a random walk time series $\{ Y(i)\}$. Subsequently, further multifractal analysis is performed on this time series.

(3) Divide the time series $\{ Y(i)\}$ into *m* non-overlapping intervals of length *s*, where each interval $v$ contains *s* points. Apply the least squares method to perform polynomial fitting on each interval using an n-order polynomial, resulting in a fitted polynomial trend value. This is defined as shown in formula (3):

3 $${\text{y}}_{\text{v}} ({i})=a_1{{i}}^k+a_2{{i}}^{k-1}+...a_{{k}}{\text{i}}+a_{{k+1}}$$

where *i*=1, 2, 3, ..., *s*, *k*=1,2, ..., 2*N*.

(4) For each interval $v$, subtract the fitted polynomial trend value $y_{v} (i)$ from the values of all points within the interval to obtain the fitting residuals. These residuals represent the local trends of the interval, reflecting changes in magnitude rather than structural changes. Calculate the mean square value of the local trend to obtain the scale function $F(v,\;s)$, as shown in formula (4):

4 $$F^{2} \;(v,\;s) = \frac{1}{s}\sum\limits_{i = 1}^{s} {\{ Y[(v - 1)s + i] - y_{v} (i)\}^{2} }$$

(5) Since intervals contain varying numbers of points, if the time interval is short, meaning it contains fewer data points, then fast waves influence the scale function, whereas slow waves influence it when the interval is long. Therefore, when calculating the scale function, the time series is divided into segments of different lengths to highlight the effects of fast and slow waves in the time series, denoted as $RMS\{ ns\} (v)$. This is represented in formula (5):

5 $$RMS\;\{ ns\} \;(v) = F(v,\;s)$$

where *ns* represents the number of different time intervals, and $v$ represents an interval.

(6) Calculate the average of all intervals to obtain the q-th order fluctuation function, as shown in formula (6):

6 $$F_{q} (s) = \left\{ {\frac{1}{n}\sum\limits_{v = 1}^{{N_{s} }} {[F^{2} \;(v,\;s)]}^{\frac{q}{2}} } \right\}^{\frac{1}{q}}$$

In general, the variable *q* can take any value. For example, when $q = 2$, it corresponds to the standard DFA procedure, where the fluctuation function increases with the increase of s, in genera $s \ge m$.

(7) If the time series $\{ x_{k} \}$ exhibits long-range correlations, then the relationship described by Eq. (7) holds:

7 $$F_{q} (s)\sim s^{h(q)}$$

(8) From Eqs. (2–7), it can be observed that the fluctuation function and the number of points within each interval exhibit an exponential relationship. Taking the logarithm of both sides, as shown in Eq. (8):

8 $$\lg \;F_{q} (s) = h(q)\lg \;s + C$$

9 $$h(q)\sim q$$

The slope obtained from linear regression using Eq. (9) represents the scaling exponent $h(q)$, also known as the Hurst exponent. The Hurst exponent, proposed by H.E. Hurst, is a parameter used to determine whether a time series has temporal dependence. It is primarily used to determine whether data exhibits chaotic behavior and to detect subtle changes in the characteristics of a sequence^23^. Depending on the value of *q*, different amplitude fluctuations can be described. For instance, when *q* takes a larger value, it describes the scaling behavior of larger amplitude fluctuations, while a smaller *q* value describes the scaling behavior of smaller amplitude fluctuations. Each order corresponds to a Hurst exponent, dependent on *q*, denoted as *q*-Hurst, as shown in Eq. (9). For example, when *q* = 2, the scaling exponent $h(2)$ is known as the classical Hurst exponent.

(9) Transforming the q-th order $h(q)$ into the q-th order mass exponent $\tau (q)$ is shown in Eq. (10):

10 $$\tau (q) = qh(q) - 1$$

From Eq. (10), it can be observed that the mass exponent is related to the order and the Hurst exponent. This relationship also elucidates the connection between the *q*-Hurst exponent and the mass exponent. The mass exponent is typically used for calculating singularity exponents and singularity dimensions. The mass exponent is a nonlinear function; if it were a linear function, it would not indicate multifractal but rather a monofractal process.

The singularity exponent $\alpha$ is used to describe the different degrees of singularity within intervals, and it is inversely proportional to the singularity. The fractal singularity dimension $f(\alpha )$ is based on singularity strength and directly reflects the fractal dimension of singularity exponents, representing the density of the distribution of corresponding singular values.

(10) The singularity index $\alpha$ and $q$-order singularity dimension $f(\alpha )$ are calculated by Eqs. (11) and (12):

11 $$\alpha = \tau^{\prime}(q)$$

12 $$f(\alpha ) = q\alpha - \tau (q)$$

$\alpha$ reflects the different degree of singularity of each interval, and $f(\alpha )$ reflects the fractal dimension of $\alpha$ with singularity index. The $\alpha { - }f(\alpha )$ curve is generally called a multifractal spectrum curve, and its shape is a unimodal arch.

13 $$\Delta \alpha = \alpha_{\max } - \alpha_{\min }$$

Formula (13) represents the width of the fractal spectrum, representing the difference between the maximum and minimum probabilities. The size of the width not only reflects the uniformity of the distribution of time series, but also reflects the order of the brain to receive and process information and the strength of the EEG neural activity. The larger the width of the singular spectrum, the more uneven the distribution, and the larger the multifractal intensity^24,25^.

(11) The maximum value $f_{\max } (\alpha )$ of the multifractal spectrum is obtained at $\alpha_{0}$ and has $f_{\max } (\alpha ) = f(\alpha_{0} ) = 1$. At $\alpha_{0}$, the least square method can be used to fit the multifractal spectrum $f(\alpha )$ to obtain Eq. (14).

14 $$f(\alpha ) = {\text{A}}(\alpha - \alpha_{0} )^{2} + {\text{B}}(\alpha - \alpha_{0} ) + 1$$

where A and B can be obtained by fitting the multifractal spectral curve. B is used to describe the degree of asymmetry of the multifractal spectral curve. When B = 0, the multifractal spectral curve is completely symmetric. When B > 0, it indicates that the multifractal spectral curve is skewed to the left. When B < 0, the multifractal spectral curve is skewed to the right. Previous studies have shown that for time series with large fluctuations and complex changes, the left-biased spectrum, that is, when B > 0, shows more abundant multifractal structures^26^.

### Institutional review board statement

The study was conducted according to the guidelines of the Declaration of Helsinki, and approved by the Scientific Research Ethics and Technology Safety committee of Northeast Electric Power University.

### Informed consent

Informed consent was obtained from all subjects involved in the study.

## Results

### Subjective statistics

In this experiment, subjects' subjective evaluation of the shape of charging piles was recorded by the converter (Fig. 2C). Subjects press the left button of the converter to indicate liking, otherwise, they press the right button. Table 1 shows the subjective evaluations of all subjects recorded by the converter.

**Table 1:** The subject's subjective emotional response to different shapes of charging piles.

	Charging piles with curved appearance design				Charging piles with square appearance design			
	Like		Dislike		Like		Dislike	
	Number of pictures	Proportion (%)	Number of pictures	Proportion (%)	Number of pictures	Proportion (%)	Number of pictures	Proportion (%)
Subject 1	47	94	3	6	8	16	42	84
Subject 2	45	90	5	10	7	14	43	86
Subject 3	49	98	1	2	3	6	47	94
Subject 4	50	100	0	0	2	4	48	96
Subject 5	47	94	3	6	5	10	45	90
Subject 6	48	96	2	4	3	6	47	94
Subject 7	48	96	2	4	5	10	45	90
Subject 8	46	92	4	8	2	4	48	96
Subject 9	50	100	0	0	3	6	47	94
Subject 10	45	90	5	10	7	14	43	86
Subject 11	49	98	1	2	3	6	47	94
Subject 12	45	90	5	10	5	10	45	90
Subject 13	50	100	0	0	3	6	47	94
Subject 14	50	100	0	0	9	18	41	82
Subject 15	48	96	2	4	2	4	48	96
Subject 16	47	94	3	6	2	4	48	96
Subject 17	49	98	1	2	8	16	42	84
Subject 18	48	96	2	4	5	10	45	90
Subject 19	50	100	0	0	5	10	45	90
Subject 20	47	94	3	6	3	6	47	94
The average	47.9	95.8	2.1	4.2	4.5	9	45.5	91

It can be seen from Table 1 that most subjects prefer charging piles with curved design, while most subjects are indifferent to charging piles with square design, that is, most subjects do not like this type of charging piles.

### The generalized Hurst exponent

**Figure 3:**
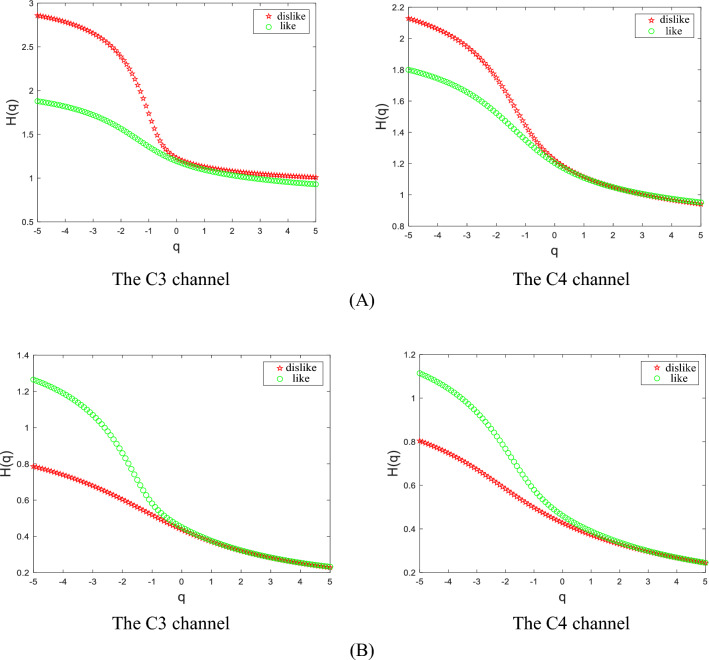
Hurst exponent of subject. (**A**) The Hurst exponent of the *θ* sub-bands in C3 and C4 channels; (**B**) The Hurst exponent of the *β* sub-bands in C3 and C4 channels.

In this experiment, EEG *θ* rhythm signals and EEG *β* rhythm signals in C3 and C4 channels of subjects were extracted, and the generalized Hurst exponent was obtained by the MF-DFA method, as shown in Fig. 3.

Literature^27^ shows that for a time series signal, if its Hurst exponent shows a decreasing trend with the growth of *q*, the time series signal has a significant multiscale property. In Fig. 3, *H*(*q*) is a nonlinear function about *q.* As* q* grows, the value of *H*(*q*) shows a decreasing trend. It shows that the physiological time series of *θ* rhythm signal and β rhythm signal have obvious multiscale properties^27^. Therefore it is ideal to study the EEG *θ* rhythm signal and EEG *β* rhythm signal by the method of multiple fractals. We can also see from the figure that the value of *H*(*q*) tends to stabilize when *q* is less than − 5 or more than 5. For different *q* there exist different exponential powers, i.e., there exist different power-law autocorrelations. Moreover, the values of *H*(*q*) are not all equal to 0.5, and *H*(*q*) = 0.5 is a random Brownian motion, which indicates that the studied EEG *θ* rhythm signal and EEG *β* rhythm signal have non-smooth characteristics. So the study with the MF-DFA method can portray the characteristics of physiological time series more precisely.

As can be seen from Fig. 3, when subjects showed negative emotions such as dislike, indifference, and disinterest in the appearance of charging piles, the Hurst exponent of *θ* rhythm signal in C3 and C4 channels has a large range, and the corresponding EEG signals of *θ* rhythm have a large uneven distribution, complexity, and signal volatility. However, when the subjects showed positive emotions such as liking and interest in the appearance of the charging piles, the Hurst exponent value range of the *θ* rhythm signal in C3 and C4 channels was small, and the corresponding *θ* rhythm EEG signal distribution, complexity and signal volatility were small. On the contrary, for the range of Hurst exponent values of EEG *β* rhythm signal in C3 and C4 channels, when the subjects showed negative emotions such as dislike, indifference, and disinterest in the shape of the charging piles they saw, the range of Hurst exponent values of the *β* rhythm signal in the C3 and C4 channels was smaller, and at this time the corresponding unevenness in the distribution of the *β* rhythm EEG signal, their complexity, and the signal fluctuation were smaller; whereas when the subjects showed positive emotions such as liking and interest in the shape of the seen charging pile, the Hurst index of the *β* rhythm signal in the C3 and C4 channels took a larger range of values, and at this time, the corresponding *β* rhythm EEG signal had a larger degree of unevenness of distribution, complexity and signal volatility. Therefore, the generalized Hurst exponent obtained by the MF-DFA method can effectively judge the subjects' emotional cognition of the charging pile shapes.

### Multifractal spectrum

**Figure 4:**
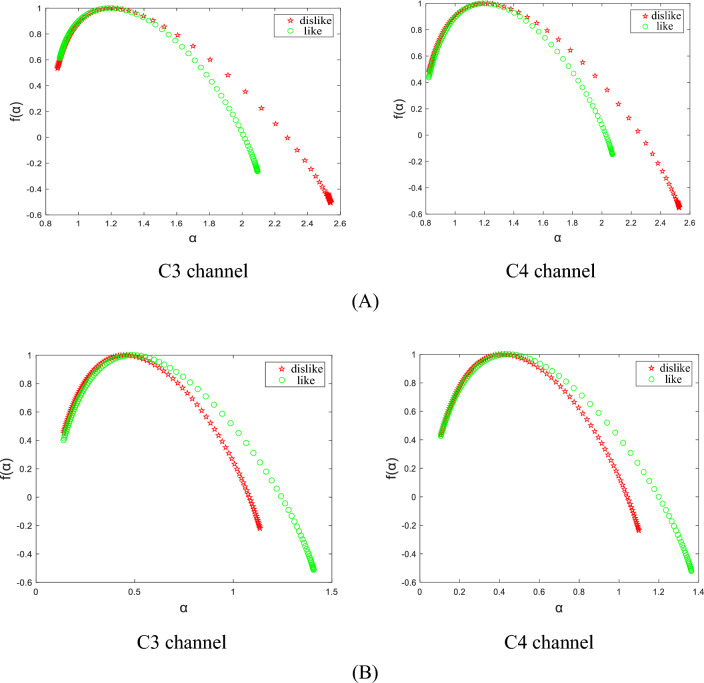
Multifractal spectrum. (**A**) The Multifractal spectrum of the *θ* sub-band in C3 and C4 channels; (**B**) The Multifractal spectrum of the *β* sub-band in C3 and C4 channels.

In this experiment, the average multifractal spectrum of the EEG signals corresponding to the two different emotions of the subjects was obtained by the MF-DFA method, as shown in Fig. 4. In the figure, *α* represents the *q*-order singularity index, and *f* (*α*) denotes the number of singular dimensions under the corresponding *α*. The singularity index reflects the different degrees of singularity in each interval, the singularity dimension reflects the fractal dimension with the singularity index, and the two constitute the multifractal spectrum curve. The singular dimension is different from the general dimension in that it is an important parameter for describing time series with multiple fractals. The size of the fluctuations in the segments that deviate from the average structure of the multifractal is defined as the width of the multifractal spectrum. The width of the multifractal spectrum is written as the difference between the maximum and minimum values of *α*. The width of the multifractal spectrum has also been widely used as a parameter to measure the strength of multifractals. It has been found that the larger the width of the multifractal spectrum, the greater the multifractal intensity, indicating a more uneven distribution and a higher complexity of the time series^25,26^.

As can be seen from Fig. 4, when the subjects showed negative emotions such as dislike, indifference, and disinterest in the shape of charging piles they saw, the multifractal spectrum width of the *θ* rhythm signal in the C3 and C4 channels were larger, and at this time the corresponding *θ* rhythm EEG signal had a greater degree of unevenness of distribution, complexity, and signal fluctuation; whereas when the subjects showed positive emotions such as liking, interest, and so forth in the shape of the charging piles they saw, the multifractal spectrum width of the *θ* rhythm signal in the C3 and C4 channels were smaller, and at this time the corresponding *θ* rhythm EEG signal had a lower degree of unevenness of distribution, complexity, and signal fluctuation. On the contrary, when the subjects showed negative emotions such as dislike, indifference, and disinterest in the shape of the charging piles seen, the multifractal spectrum width of the *β* rhythm signal in the C3 and C4 channels was smaller, and at this time the corresponding *β* rhythm EEG signal showed less unevenness in the degree of distribution, complexity, and signal fluctuation; and when the subjects showed positive emotions such as liking and interest in the shape of the charging pile they saw, the multifractal spectrum width of the* β* rhythm signal in the C3 and C4 channels was larger, and at this time, the corresponding *β* rhythm EEG signal had a greater degree of unevenness in distribution, complexity, and signal volatility. Therefore, the multifractal spectrum obtained by the MF-DFA method can effectively judge the emotional cognition differences of different charging pile shapes.

**Figure 5:**
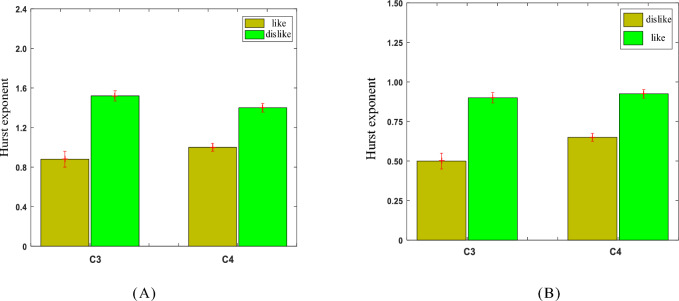
Hurst exponent. (**A**) The Hurst exponent of the *θ* sub-bands in C3 and C4 channels; (**B**) The Hurst exponent of the *β* sub-band in C3 and C4 channels.

**Figure 6:**
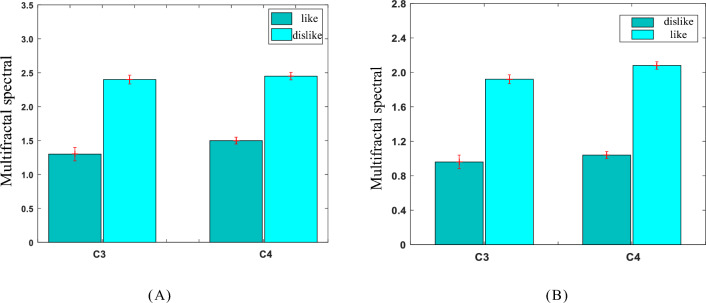
Multifractal spectrum width. (**A**) The Multifractal spectrum width of the *θ* sub-bands in C3 and C4 channels; (**B**) The Multifractal spectrum width of the *β* sub-band in C3 and C4 channels.

The mean and standard deviation of the generalized Hurst exponent and multifractal spectrum width for the EEG signals corresponding to the two types of emotions for the 20 subjects are shown in Figs. 5 and 6.

As can be seen from Figs. 5 and 6, the Hurst index and multifractal spectrum width of *θ* rhythm in C3 and C4 channels of subjects were significantly smaller than those of subjects facing pictures of charging piles they disliked. On the contrary, the Hurst exponent and multifractal spectrum width of *β* sub-band in C3 and C4 channels of subjects were significantly larger than those of subjects facing pictures of charging piles they disliked. It can be concluded that the multifractal features of the two different types of emotional cognition generated by subjects on the shape of charging piles are contrasted significantly, and the two types of emotional cognition features are significantly different (P < 0.05), i.e., the use of the multifractal algorithm can effectively differentiate between the different cognitions of the subjects on the shape of charging piles.

In summary, based on the characteristics of human EEG signals, the emotional differences caused by different charging piles can be effectively distinguished. The method of this study is helpful to reduce the influence of human subjective factors on product evaluation and provide a more objective reflection of users' evaluation of product design. Traditional product evaluation methods usually rely on subjective questionnaires, which are easily affected by factors such as participants' personal preferences, social expectations and answer bias. In contrast, the use of objective measurement data such as EEG features can more directly capture people's actual emotional response to the appearance of the product, avoiding the impact of subjective bias and memory distortion on the evaluation results.

## Discussion

### Previous studies and this study

Users' satisfaction with product appearance can be obtained through a subjective questionnaire survey. Product shape designers can improve product shape by users' satisfaction with the product, so that it is more in line with people's aesthetic senses^28^. However, a subjective questionnaire survey is sometimes susceptible to the subjective feelings of the subjects, and the evaluation of product appearance is not comprehensive, such as the lack of human emotion recognition^29–31^. Therefore, it is necessary to analyze and investigate people's emotional cognition of product appearance from an objective perspective. Studies have shown that different product shapes will stimulate people's brain nerves to different degrees, resulting in different cognitive differences^29–35^. Zhang's research shows that different shapes of the same product will produce different neural stimulation to people, and these differences are particularly obvious in the EEG characteristics^36^. Desmet P.M.A.'s research shows that different product design shapes will produce different emotional responses to human body, and this difference can be reflected in human physiological signal characteristics^37,38^. Qiu Kai's research shows that different product shapes can stimulate people's visual nerves to different degrees, and the difference of eye movement features is very obvious^37^. In this study, EEG features were used to analyze the differences in human emotion recognition caused by the appearance of charging piles.

When the human body produces different emotions, the corresponding brain regions will produce different EEG signal characteristics. According to Wang's research, different car shapes will produce different emotional cognition, which is specifically manifested in the different activity levels of *β* waves in the frontal and central regions of the brain and* α* waves in the top and occipital lobes of the brain^18^. Andreas et al. analyzed the cognitive differences caused by different shapes by studying the EEG features of the frontal and occipital lobes of the brain, and the results showed that the EEG features of these two regions could effectively distinguish several typical shapes of objects^38^. Because the human body has different cognitions of different product shapes, this paper uses a multifractal algorithm to analyze the difference in EEG characteristics caused by this cognitive difference between the occipital and central regions of the brain. As can be seen from Table 1, Figs. 5 and 6, different shapes of charging piles will cause people’s different emotional cognition, and different emotional cognition will produce significantly different characteristics in specific brain regions of human beings. For example, when subjects see the shape of charging piles they like or dislike, there is a significant difference in the EEG activity between the central and occipital regions of the brain. In addition, this method is different from the traditional subjective survey method used in product shape design. It is a kind of physiological and emotional characteristic of human beings, which is not affected by subjective factors of subjects, and can objectively reflect people's emotional cognition of product shape design.

### Limitations and future research lines

The purpose of this study was to analyze the differences in emotional cognitive characteristics caused by different shapes of charging piles by using the EEG characteristics of subjects. In the experiment, charging piles with curved shape design and square shape design were selected for comparative analysis, while charging piles with other shapes, such as those with square and curved line features, were not included in the experimental scope. In our future research, more types of charging pile shapes will be involved, so as to further study the differences in customer EEG characteristics corresponding to different charging pile shapes, and establish the differences in the impact of charging pile shapes on users’ emotional cognition. Finally, objective feedback on the shape design of the charging pile is realized, and further optimization of the shape of the charging pile is realized.

## Conclusion

This study uses the multifractal method to calculate the EEG signal characteristics corresponding to the brain regions (occipital region and central region) where human emotional nerves are active, and analyzes the cognitive differences caused by the two different shapes (curved appearance design and square appearance design) of charging piles. The results show that different charging pile shapes will cause people's different emotional cognition, and different emotional cognitions in the human brain central region and occipital region of the EEG signal active degree have obvious differences. In addition, the method used in this study is different from the traditional subjective survey method, and it is a kind of physiological emotional characteristics of human beings, without being influenced by the subjective factors of the subjects, and can more objectively reflect the people’s emotional cognition of the product shape design. Using this objective evaluation method based on physiological signals can provide more reliable guidance for product design and promotion. Designers and marketers can optimize the appearance design of products according to these objective data to better meet the emotional cognitive needs of users, so as to improve the market competitiveness and promotion effect of products. At the same time, this method can also help enterprises more accurately understand the preferences and reactions of consumers, guide the formulation of future product development and marketing strategies, and bring greater commercial success to enterprises.

### Supplementary Information

Supplementary Information.

## Data Availability

The effective data email: wangfuwangfeixue@163.com.
