# Supplementary material for: The effect analysis of shape design of different charging piles based on Human physiological characteristics using the MF-DFA
Source: Sci Rep. 2024 Apr 9;14:8345. doi: 10.1038/s41598-024-59147-8 (PMC11004129; doi:10.1038/s41598-024-59147-8)
Supplement: Supplementary file 1 — Supplementary Information. [file 41598_2024_59147_MOESM1_ESM.docx]

**Appendix I**

| Sequence number | Picture of Class MDCSS charging pile | Given name | Type number | Production company |
| --- | --- | --- | --- | --- |
| 1 | **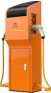** | New energy charging pile | TSC-EVCP-RE | Beijing Tai Sheng Chang Technology Co., LTD |
| 2 | **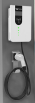** | Wall charging pile | HCC-WBCP-849 | Hechuang intelligent Technology Co., LTD |
| 3 | **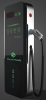** | Vertical cabinet type single gun 7KW AC charging pile | RJ-HCD-MC7 | Shandong Ruijie intelligent equipment Co., LTD |
| 4 | **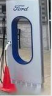** | Ford Supercharger | FC-SCP-BF9 | Ford |
| 5 | **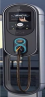** | Intelligent AC charging pile | JMPD-ICC-Y7 | Beijing Jian Meng product design company |
| 6 | **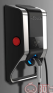** | Wall mounted AC charging pile | BLT-WACCP-J9 | Shenzhen Bolatu Industrial design Co., LTD |
| 7 | **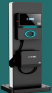** | Electric vehicle charging pile | BF-EDC-T3 | Shenzhen white fox industrial design company |
| 8 | **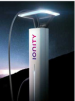** | IONITY super fast charging pile | IHK-FCDC-D30 | IONITY |
| 9 | **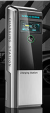** | New energy equipment charging pile | DHWL-NEDC-XZ | Donghua Future Industrial Design Company |
| 10 | **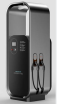** | EV Charger | APL-EVC-37 | Guangdong Aipula new energy Technology Co., LTD |
| 11 | **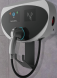** | Mini AC charging pile | HYNC-MACC-6 | Hua Yi can technology Co., LTD |
| 12 | **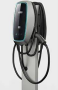** | ABBEVinnTA-GB-W7-Z5-BR-AE | ABB-GBC-75-ZK-42-AE | ABB (China) Limited |
| 13 | **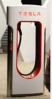** | Tesla Supercharging pile | TSLA-SCCP-A3 | TSLA |
| 14 | **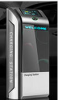** | Wall mounted AC charging pile | DHWL-WACCP-FA9 | Shandong Donghua future Industrial design Co., LTD |
| 15 | **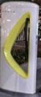** | Xiaopeng automobile super charging pile | YST-SCCP-R6 | Easy Special LTD |
| 16 | **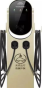** | Ac charging pile | ALD-ACCP-M63 | Hefei Anlunjie new energy Technology Co., LTD |
| 17 | **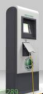** | Electric vehicle fast charging pile | HRC-FSCCP-V | Beijing Hao Ruichang Technology Co., LTD |
| 18 | **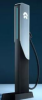** | Nio Power overfilled pile | NIO-CSCP-H9 | Nio Auto Technology Co., LTD |
| 19 | **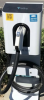** | Special charging pile | TLC-DCP-KL | Special call new energy Co., LTD |
| 20 | **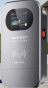** | Star AC charging pile | RFV-ACCP-753 | Nanjing Ruifanda new energy Technology Co., LTD |
| 21 | **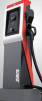** | EVDC-ZD series DC charging pile | YSTG-EVDCZD-I97 | East Group Co.,Ltd. |
| 22 | **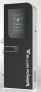** | New energy vehicle charging pile | HYNC-NECP-LP34 | Shenzhen Hua Yi can technology Co., LTD |
| 23 | **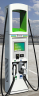** | 30KW AC pile | XDZ-ACCP-F35 | Xiangding intelligent technology Co., LTD |
| 24 | **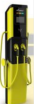** | Floor type DC charging pile | BLT-DCP-A52 | Shenzhen Bolatu Industrial design Co., LTD |
| 25 | **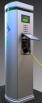** | Dc charging pile for electric vehicles | WDD-DCP-369 | Sichuan Wolde Electric Co., LTD |
| 26 | **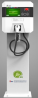** | Ankerui DC charging pile | AKR-DCP-A78 | Acrel Co., Ltd. |
| 27 | **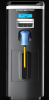** | Vertical charging pile | SHD-UCP-ZE9 | Shenzhen Shenghong Electric Co., LTD |
| 28 | **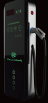** | Vertical cabinet type single gun 7KW AC charging pile | RJ-HCD-M16 | Shandong Ruijie intelligent equipment Co., LTD |
| 29 | **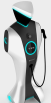** | Charging robot | CNCC-RB-J3 | Zhongnongcong Technology Co., LTD |
| 30 | **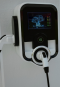** | National standard wall hanging AC 7KW charging pile | WBS-WBACCP-165 | Weibast |
| 31 | **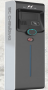** | eCharger charging pile | PSDL-ECP-489 | Nanjing Pusdir Electronic Technology Co., LTD |
| 32 | **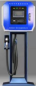** | Wall mounted charging pile | HCC-WACP-O2 | Hechuang intelligent Technology Co., LTD |
| 33 | **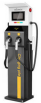** | Vertical double gun charging pile | SHD-DCP-245 | Shenzhen Shenghong Electric Co., LTD |
| 34 | **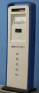** | Slow charging pile | LZKT-SCCP-U56 | Nanjing Green Exhibition Technology Co., LTD |
| 35 | **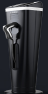** | Single gun vertical charging pile | HJL-UCP-341 | Hongjiali New energy Co., LTD |
| 36 | **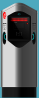** | Intelligent charging pile | KDI-ICCP-33 | CSG Smart Science&Technology Co., Ltd. |
| 37 | **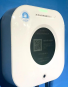** | C2 intelligent AC charging pile | ZC-ACCP-C2-78 | Beijing Zhichong Technology Co., LTD |
| 38 | **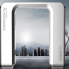** | An Yue charging pile | SAG-ACCP-123 | Shanghai SAIC Anyue charging Technology Co., LTD |
| 39 | **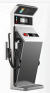** | Floor type charging pile | BLT-FDCP-HF3 | Bolatu |
| 40 | **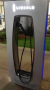** | lincoln charging pile | LCN-CCP-WQ7 | Lincoln Motor Company |
| 41 | **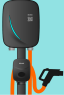** | Wall charging pile | BHF-WACP-YT1 | Shenzhen white fox industrial design company |
| 42 | **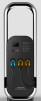** | AC charging pile | BGT-ACCP-6 | Boguang Electrical technology Co., LTD |
| 43 | **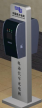** | All-in-one charger | TYK-CCP-RE | Shenzhen Tengye technology Co., LTD |
| 44 | **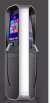** | Saic Anyue charging pile | SAG-ACCP-J | Shanghai SAIC Anyue charging Technology Co., LTD |
| 45 | **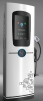** | Direct flow charging pile | SHD-DCDCP-24 | Shenzhen Shenghong Electric Co., LTD |
| 46 | **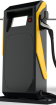** | Shared charging pile | TLD-SCCP-W1 | Special call new energy Co., LTD |
| 47 | **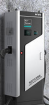** | 90-150KW DC dual gun charging pile | OE-DCDCP-D2 | Shaanxi Eurasia Electric Appliance Co., LTD |
| 48 | **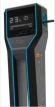** | Dc charging pile | DYC-DCDCP-G87 | Daye Product Design Co., LTD |
| 49 | **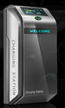** | Wall mounted AC charging pile | SD-DHWL-WACCP-5 | Shandong Donghua future Industrial design Co., LTD |
| 50 | **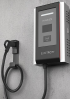** | Wall mounted charging pile | ZNEN-WACP-H12 | Zhongwei Energy Technology Co., LTD |

**Appendix II**

| Sequence number | Picture of Class MDSSS charging pile |  | Given name | Type number | Production company |
| --- | --- | --- | --- | --- | --- |
| 1 | 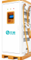 |  | Medium-sized home charging piles | GB/T18487.3-2015 | Anhui Zhongneng New Energy Technology Co., Ltd |
| 2 | 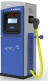 |  | Single-shot DC pile | EV-S90S90PU | Jiangsu Chunchen Cable Co., Ltd |
| 3 | 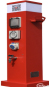 |  | Smart hydropower piles | GB/T20234.2-2011 | Xi'an Gongtong Energy Co., Ltd |
| 4 | 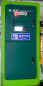 |  | DC charging pile | GZXC-131 | State Grid Corporation of China |
| 5 | 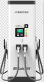 |  | Column-type double-gun charging pile | GB/T20234.1-2011 | Hongjiali New Energy Company |
| 6 | 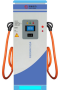 |  | DC charging piles for electric vehicles | GB/T 7930-2015 | Beijing Qiyuan New Energy Technology Co., Ltd |
| 7 | 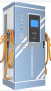 |  | All-in-one car charging pile | GB/T18487.1-2015 | Shenzhen Encore Technology Co., Ltd |
| 8 | 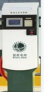 |  | EV charging piles | GB/T20234.2-2011 | Suzhou Rainbow Electric Co., Ltd |
| 9 | 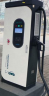 |  | Vertical car charging piles | DFYG-DC-026 | Oriental Yaguang (Beijing) Technology Co., Ltd |
| 10 | 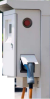 |  | Small EV charging piles | GB/T20234.3-2015 | China Southern Power Grid Co., Ltd |
| 11 | 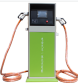 |  | Integrated charging pile for electric vehicles | ZW-AC-001 | Henan Zhiwang Power Equipment Co., Ltd |
| 12 | 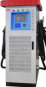 |  | Vertical double-gun charging pile | HN-DC-003 | Huineng New Energy (Shanghai) Co., Ltd |
| 13 | 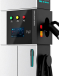 |  | All-in-one charging pile | GB/T27930-2015 | Star Charging Co., Ltd |
| 14 | 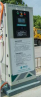 |  | EV charging piles | GB/T28930-2015 | Weizhong New Energy Co., Ltd |
| 15 | 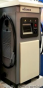 |  | DC double-gun charging pile | GB/T27940-2015 | Hongda Charging Co., Ltd |
| 16 | 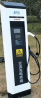 |  | AC single-gun charging pile | MS101-500 | Shenzhen Pole Number Charging IoT Technology Co., Ltd |
| 17 | 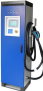 |  | DC charging piles for electric vehicles | YST-DC-009 | East Group Co., Ltd |
| 18 | 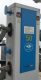 |  | Vertical double-gun charging pile | ZGH-AC-200 | CGN New Energy Vehicle Technology Co., Ltd |
| 19 | 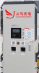 |  | All-in-one vertical charging pile | MS201-600 | The car came to the company |
| 20 | 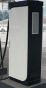 |  | Electric charging piles | GX-AC-200 | Gotion Hi-Tech Co., Ltd |
| 21 | 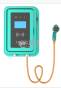 |  | Double rectangular wall-mounted AC single-gun charging pile | KMY-AC-200 | Muyang Titan Control Technology Co., Ltd |
| 22 | 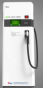 |  | Vertical single-gun pile | YL-AC-40 | E-Call Limited |
| 23 | 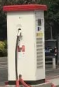 |  | Double-gun vertical charging pile | HX-DC-200 | Huaxing Power Company Limited |
| 24 | 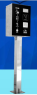 |  | Convenient small charging pile | ZD-AC-400 | CLP Holdings Limited |
| 25 | 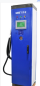 |  | EV charging piles | GB/T27832-2016 | HC Semitek Energy Co., Ltd |
| 26 | 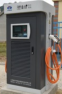 |  | Integrated charging pile for electric vehicles | BT-AC-200 | PATEO New Energy Co., Ltd |
| 27 | 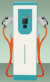 |  | Vertical double-gun charging pile | LH-D-400 | Longfor Charging Co., Ltd |
| 28 | 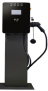 |  | Small vertical charging piles | BT-AC-200 | PATEO New Energy Co., Ltd |
| 29 | 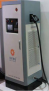 |  | DC fast charging pile | KP-DC-400 | Zhongke Puhui Co., Ltd |
| 30 | 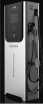 |  | EV charging piles | MS105-700 | Huasheng Charging Co., Ltd |
| 31 | 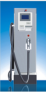 |  | Vertical and convenient charging pile | SF-AC-200 | Sanfeng New Energy Co., Ltd |
| 32 | 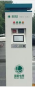 |  | All-in-one charging pile | HY-AC-200 | Hengyuan New Energy Co., Ltd |
| 33 | 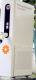 |  | Small vertical charging piles | GB/T27932-208 | Hongda Charging Co., Ltd |
| 34 | 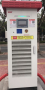 |  | DC charging piles for electric vehicles | TH-DC-200 | Tianhe Lianchuang Limited |
| 35 | 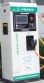 |  | Vertical single-gun charging pile | HD-AC-200 | Evergrande New Energy Co., Ltd |
| 36 | 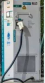 |  | AC all-in-one car charging pile | JD-AC-400 | Judian New Energy Co., Ltd |
| 37 | 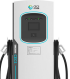 |  | Small dual-gun car charging pile | GB/T27732-2015 | ZTE New Energy Co., Ltd |
| 38 | 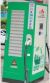 |  | Vertical double-gun DC charging pile | HC-AC-200 | HC Semitek Energy Co., Ltd |
| 39 | 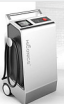 |  | Portable small charger | PS-DC-200 | Pengshuang New Energy Co., Ltd |
| 40 | 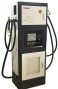 |  | Dual-gun integrated charging pile | YBX-AC-200 | UBTECH Co., Ltd |
| 41 | 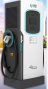 |  | Intelligent dual-gun vertical charging pile | JC-DC-120-2 | Hangzhou State-controlled Electric Power Technology Co., Ltd |
| 42 | 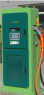 |  | DC charging pile | GB-DC-200 | State Grid Corporation of China |
| 43 | 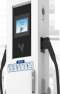 |  | Convenient charging pile for two guns | DK-AC-200 | Dekar Internet Limited |
| 44 | 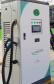 |  | All-in-one single-gun charging pile | GB/T27932-2015 | Green Action New Energy Co., Ltd |
| 45 | 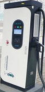 |  | EV charging piles | YL-AC-400 | Yealink New Energy Co., Ltd |
| 46 | 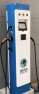 |  | Vertical double-gun intelligent charging pile | HS-DC-200 | Huasheng Charging Co., Ltd |
| 47 | 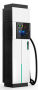 |  | All-in-one single-gun charging pile | YK-AC-400 | E-Point Charging Co., Ltd |
| 48 | 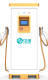 |  | Small dual-gun charging pile | GX-AC-200 | Gotion Hi-Tech Co., Ltd |
| 49 | 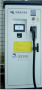 |  | Medium-sized single-gun electric vehicle charging pile | WZ-AC-400 | Weizhong New Energy Co., Ltd |
| 50 | 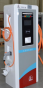 |  | Vertical double-gun charging pile | JRW-DC-400 | Jenova Co., Ltd |
